# Supplementary material for: Axonal RNA localization is essential for long-term memory
Source: Nat Commun. 2025 Mar 15;16:2560. doi: 10.1038/s41467-025-57651-7 (PMC11910521; doi:10.1038/s41467-025-57651-7)
Supplement: Supplementary file 1 — Supplementary Information [file 41467_2025_57651_MOESM1_ESM.pdf]

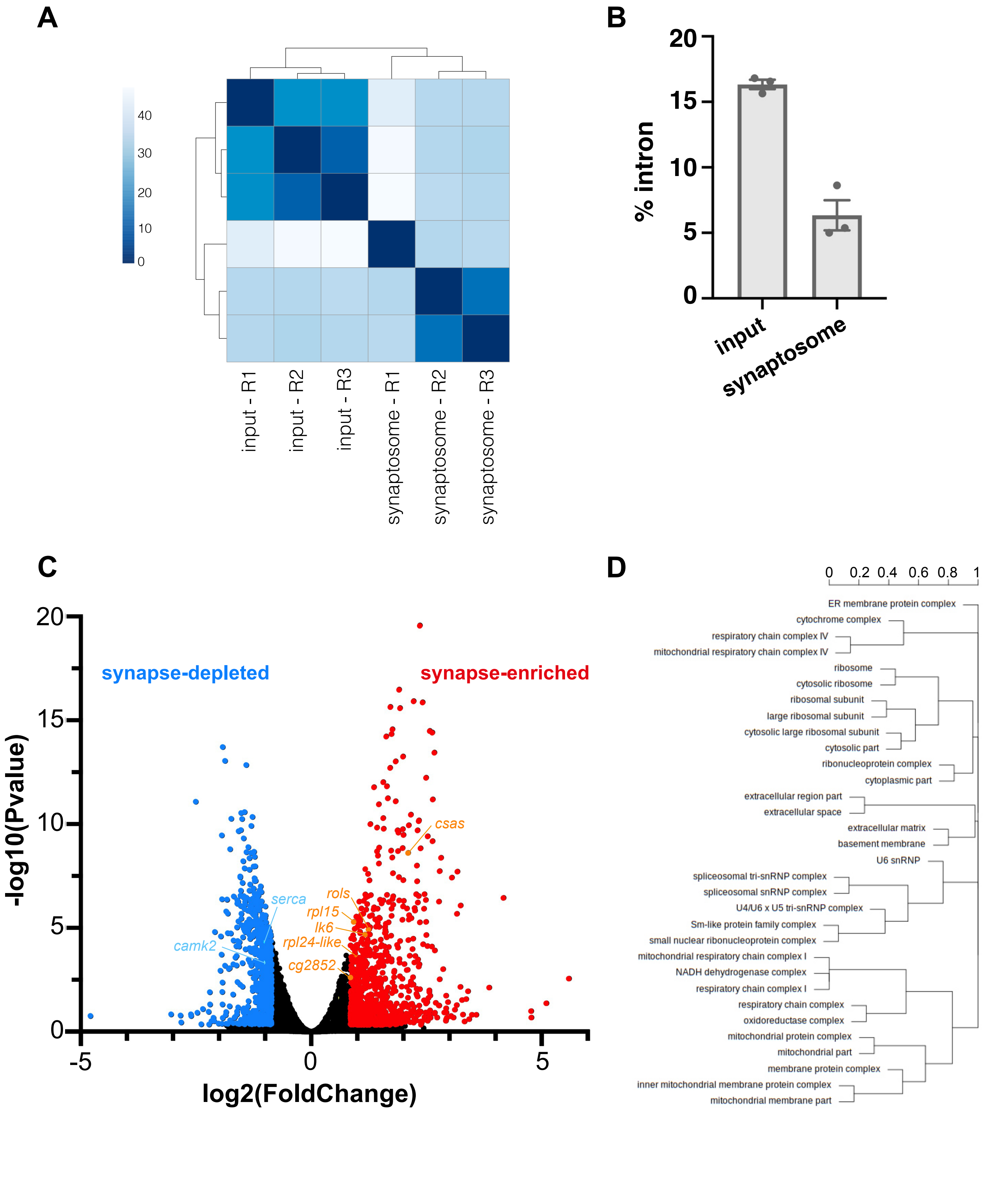

A
40
30
20
10
0
input - R1
input - R2
input - R3
synaptosome - R1
synaptosome - R2
synaptosome - R3
B
% intron
20
15
10
5
0
input
synaptosome
C
-log10(Pvalue)
20
15
10
5
0
synapse-depleted
synapse-enriched
serca
camk2
rols
rpl15
lk6
rpl24-like
cg2852
csas
-5
0
5
log2(FoldChange)
D
0 0.2 0.4 0.6 0.8 1
ER membrane protein complex
cytochrome complex
respiratory chain complex IV
mitochondrial respiratory chain complex IV
ribosome
cytosolic ribosome
ribosomal subunit
large ribosomal subunit
cytosolic large ribosomal subunit
cytosolic part
ribonucleoprotein complex
cytoplasmic part
extracellular region part
extracellular space
extracellular matrix
basement membrane
U6 snRNP
spliceosomal tri-snRNP complex
spliceosomal snRNP complex
U4/U6 x U5 tri-snRNP complex
Sm-like protein family complex
small nuclear ribonucleoprotein complex
mitochondrial respiratory chain complex I
NADH dehydrogenase complex
respiratory chain complex I
respiratory chain complex
oxidoreductase complex
mitochondrial protein complex
mitochondrial part
membrane protein complex
inner mitochondrial membrane protein complex
mitochondrial membrane part

Figure S1

Figure S1. Characterization of synaptosome RNA-seq data

(A) Heatmap of the sample-to-sample Euclidian distances. Distances were calculated from DESeq2 normalized data after regularized-logarithm transformation. Clustering of the 6 RNA-seq samples gives an overview of similarities between conditions, reflected by the color intensity. **(B)** Percentages of reads mapping to intronic regions in the initial head lysate (input) and synaptosome fraction. Individual data points correspond to replicate samples and error bars to s.e.m. **(C)** Volcano plot showing the differential enrichment of RNAs in synaptosome *vs* input fractions. Synaptosome-enriched (red; log2FC>=0.85) and synaptosome-depleted (blue; log2FC<=-0.85) RNAs are displayed in red and blue respectively. RNAs analyzed in smFISH experiments (Figures 2 and S2) are highlighted. **(D)** Hierarchical clustering of GO terms based on Jacquard similarity (see Materials and Methods). Source data are provided as a Source Data file.

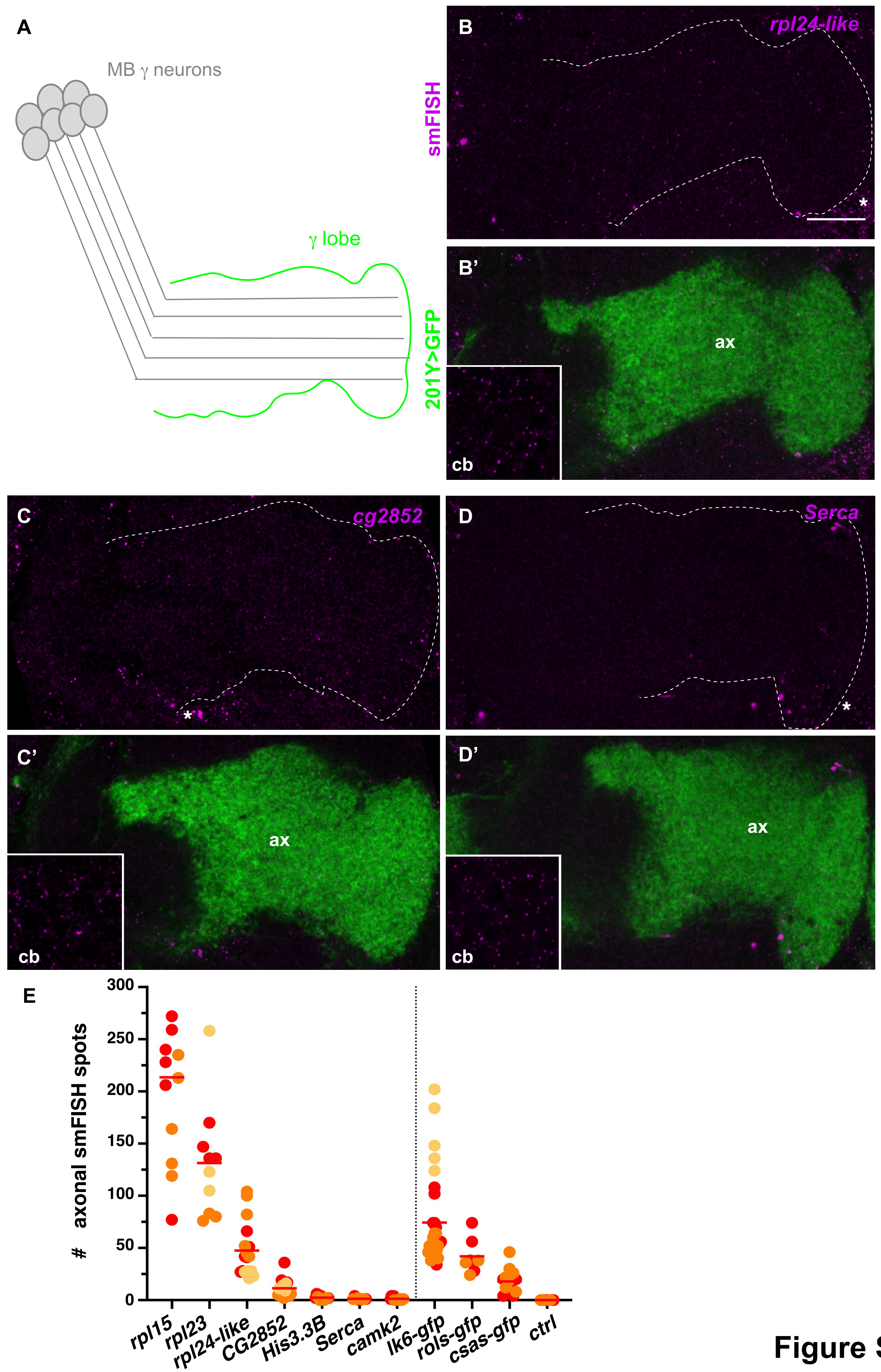

A
MB γ neurons
γ lobe
B
rpl24-like
smFISH
B'
201Y>GFP
ax
cb
C
cg2852
smFISH
C'
201Y>GFP
ax
cb
D
Serca
D'
ax
cb
E
axonal smFISH spots
300
250
200
150
100
50
0
rpl15
rpl23
rpl24-like
CG2852
His3.3B
Serca
camk2
lk6-gfp
rols-gfp
csas-gfp
ctrl

Figure S2

Figure S2. Different patterns of axonal mRNA localization

(A) Schematic representation of MB γ neurons that project their axons in the so-called medial lobe. **(B-D)** Confocal images of smFISH signals (magenta) obtained from whole mount adult brains using anti-*rpl24-like* (B), *cg2852* (C) and *Serca* (D) probe sets. Boxed areas correspond to single confocal sections of MB γ neuron cell bodies (cb). MB γ axons (ax) were labeled using the 201Y-Gal4 driver in combination with UAS-cGFP (green signals in the bottom overlay images). Asterisks indicate signals corresponding to the soma of neighboring neuronal populations. Scale bar: 10 µm. **(E)** Quantification of the number of smFISH spots detected in MB γ axons for the different transcripts analyzed. Two to three replicates were performed and data from each replicate labeled with different colors. Left: transcripts detected with gene-specific probe sets. Right: transcripts detected with anti-*gfp* probe set. Red bars correspond to average values. Source data are provided as a Source Data file.

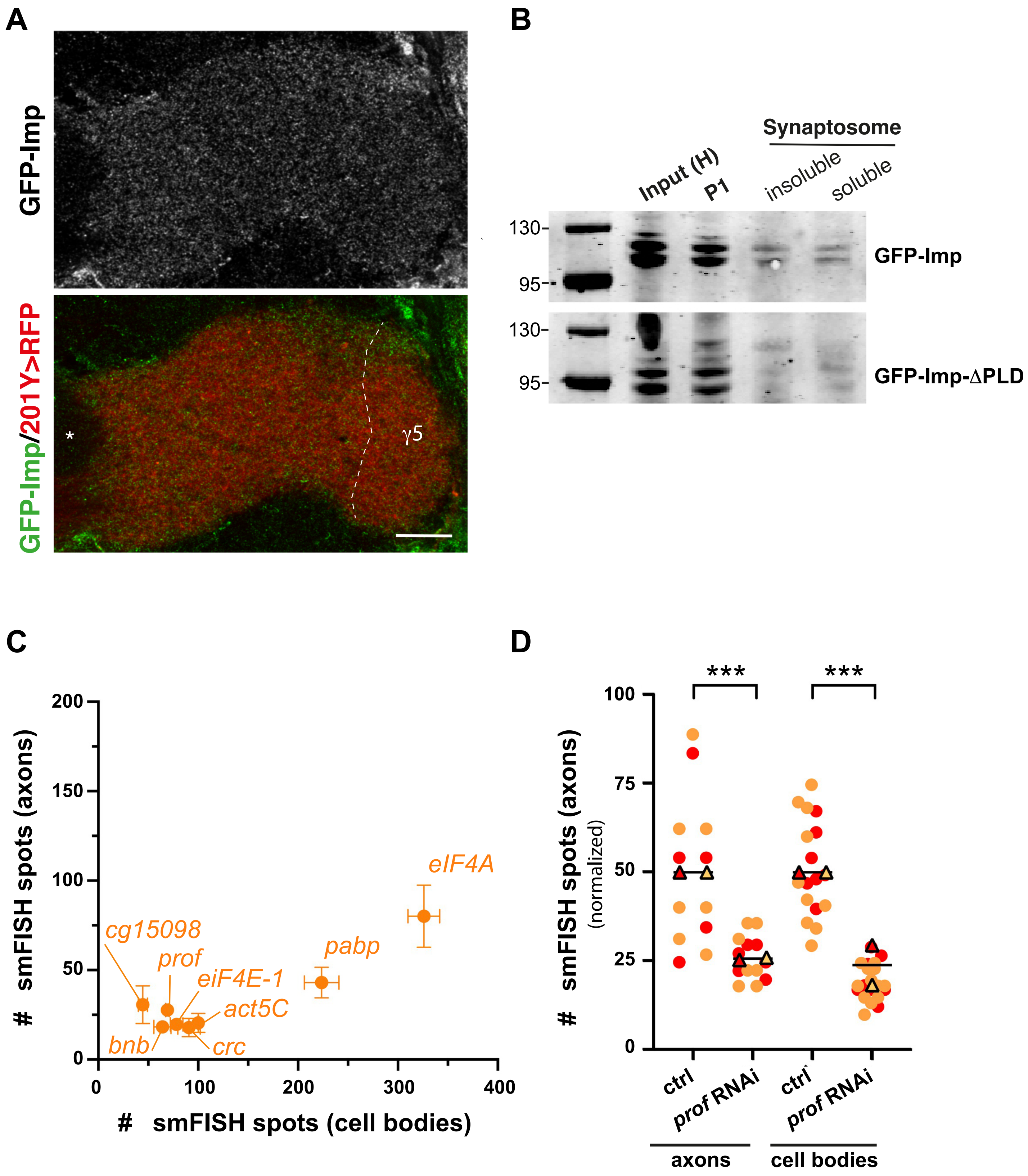
A
GFP-Imp
GFP-Imp/201Y>RFP
*
γ5
B
Synaptosome
Input (H)
P1
insoluble
soluble
130-
95-
GFP-Imp
130-
95-
GFP-Imp-ΔPLD
C
smFISH spots (axons)
smFISH spots (cell bodies)
200
150
100
50
0
0
100
200
300
400
eIF4A
cg15098
prof
pabp
eiF4E-1
act5C
bnb
crc
D
smFISH spots (axons)
(normalized)
100
75
50
25
0
***
***
ctrl
prof RNAi
ctrl
prof RNAi
axons
cell bodies

Figure S3

Figure S3. Localization of Imp and its associated RNAs in axon terminals

(A) Distribution of GFP-Imp proteins expressed from the endogenous locus (G080-GFP-Imp protein-trap insertion) in MB γ axons. GFP-Imp signal is shown in white in the upper panel and in green in the lower overlay panel. MB γ axons are labeled with CD8-RFP expressed with the 201Y-Gal4 driver (red in the lower overlay panel). The dotted white line separates the γ5 compartment from the more proximal γ compartments. The asterisk points to a region where other MB neuron types (α'β' and αβ) project their axons, highlighting that GFP-Imp is not localized there. Precise genotype: G080-GFP-Imp/Y; 201Y-Gal4, UAS-CD8-RFP/+. Scale bar: 10 µm. **(B)** Western-Blots performed on the initial head lysates (H, input) and first pellet (P1) (left) of the synaptosome preparation procedure, as well as on the synaptosome soluble and insoluble fractions (right). G080-GFP-Imp (top) and G080-GFP-Imp-ΔPLD (bottom) heads were used to prepare the lysates and anti-GFP antibodies used to detect proteins. Blots were performed from two independent replicates, with similar results. **(C)** Graph plotting the correlation between the number of smFISH spots in MB γ cell bodies (x axis) *vs* axons (y axis) for the Imp-bound mRNAs validated in Figure 3B. Data points correspond to mean values and error bars to s.e.m. Samples were collected from three replicates. **(D)** Distribution of *profilin* (*prof*) RNA smFISH spots detected in MB γ lobe (left) and MB cell bodies (right). *profilin* RNAi was expressed *via* the OK107-Gal4 driver. Data were normalized to the control flies. Two replicates were performed and the mean value of each is indicated as a triangle. Data points were color-coded based on the replicate they belong to. ***, $P<0.001$ (Mann-Whitney tests). Source data are provided as a Source Data file.

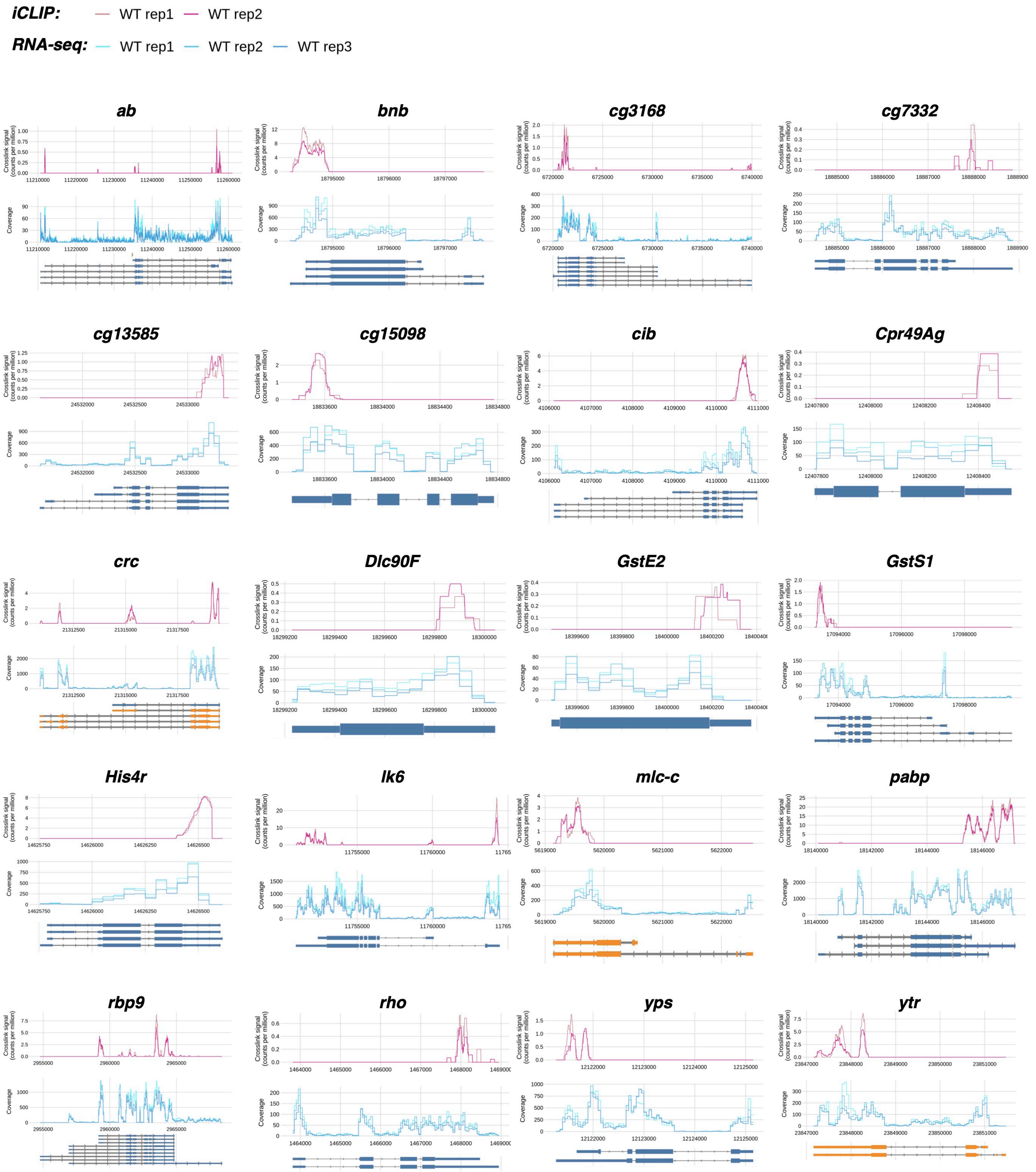
iCLIP:
WT rep1
WT rep2
RNA-seq:
WT rep1
WT rep2
WT rep3
ab
bnb
cg3168
cg7332
cg13585
cg15098
cib
Cpr49Ag
crc
Dlc90F
GstE2
GstS1
His4r
Ik6
mlc-c
pabp
rbp9
rho
yps
ytr
Crosslink signal (counts per million)
Coverage

Figure S4

Figure S4. Imp iCLIP binding profiles.

Profiles of the Imp iCLIP signals (top, two replicates) and corresponding RNA-seq signals (middle, three replicates) along the gene regions of validated Imp-bound and axonally-localized mRNAs (Figure 3A). Profiles were generated using the *clipplotr* tool [76] and its smoothening function. Intronic and exonic sequences are represented at the bottom by single lines and boxes respectively (large boxes for coding exons and smaller boxes for UTRs).

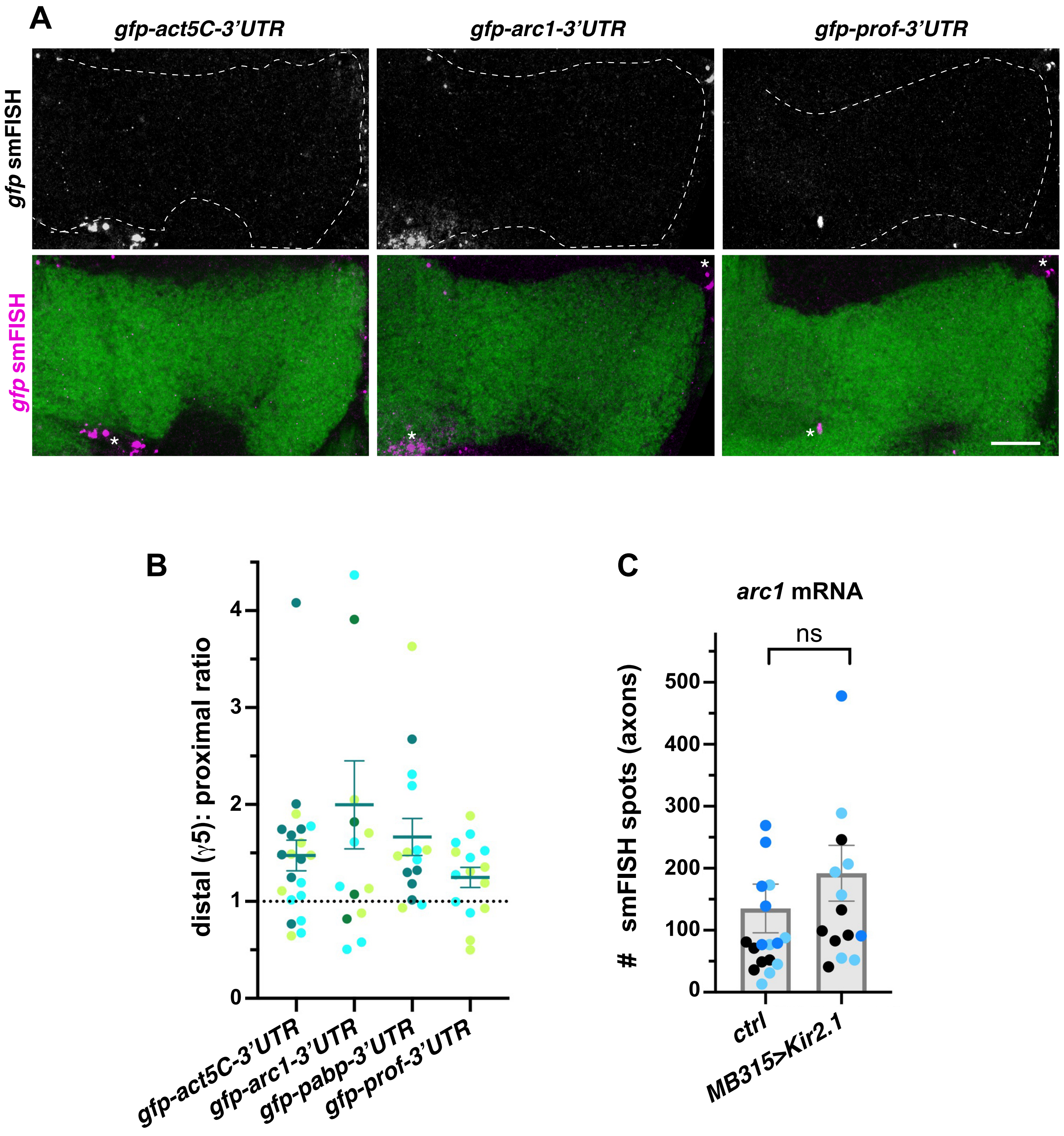
A
gfp-act5C-3'UTR
gfp-arc1-3'UTR
gfp-prof-3'UTR
gfp smFISH
gfp smFISH
B
distal (γ5): proximal ratio
0
1
2
3
4
gfp-act5C-3'UTR
gfp-arc1-3'UTR
gfp-pabp-3'UTR
gfp-prof-3'UTR
C
arc1 mRNA
ns
smFISH spots (axons)
0
100
200
300
400
500
ctrl
MB315>Kir2.1

Figure S5

Figure S5. Characterization of mRNA enrichment in MB γ5 compartment

(A) Localization of *gfp* RNAs expressed from *gfp-act5C*-3'UTR (left), *gfp-arc1*-3'UTR (middle) and *gfp-prof*-3'UTR (right) transcripts expressed using the OK107-Gal4 driver. Top: smFISH signal obtained using an anti-*gfp* probe set. Bottom: overlay between GFP protein signal (green) and *gfp* RNA signal (magenta). Asterisks point to probe aggregates. Scale bar: 10 μm. **(B)** Enrichment in the γ5 axonal compartment of *gfp* RNA expressed from different *gfp*-3'UTR reporters. The OK107-Gal4 driver was used to drive expression of UAS-*gfp*-3'UTR constructs. mRNA enrichment was quantified as the ratio of smFISH spot density (number of spots per μm^3) in the distal γ5 compartment *vs* the proximal γ2-4 compartments. Three replicates were performed and data points color-coded based on the replicate they belong to. Bars and error bars represent respectively, for each mRNA, the average and s.e.m. of all combined data points. In A,B, two independent transgenes were combined for *gfp-act5C*-3'UTR and *gfp-prof*-3'UTR reporters to increase the total number of *gfp* RNA molecules detected in axons. **(C)** Quantification of the number of *arc1* smFISH spots detected along MB γ axons. Precise genotypes: UAS-Kir2.1-GFP/+; MB247-dsRed/+ (control) and UAS-Kir2.1-GFP/+; MB247-dsRed/MB315C-Gal5 (MB315C>Kir2.1). Three replicates were performed and data from each replicate labeled with different colors. Bars and error bars represent respectively the average and s.e.m. of all combined data points. ns stands for not significant (Mann-Whitney test). Source data are provided as a Source Data file.

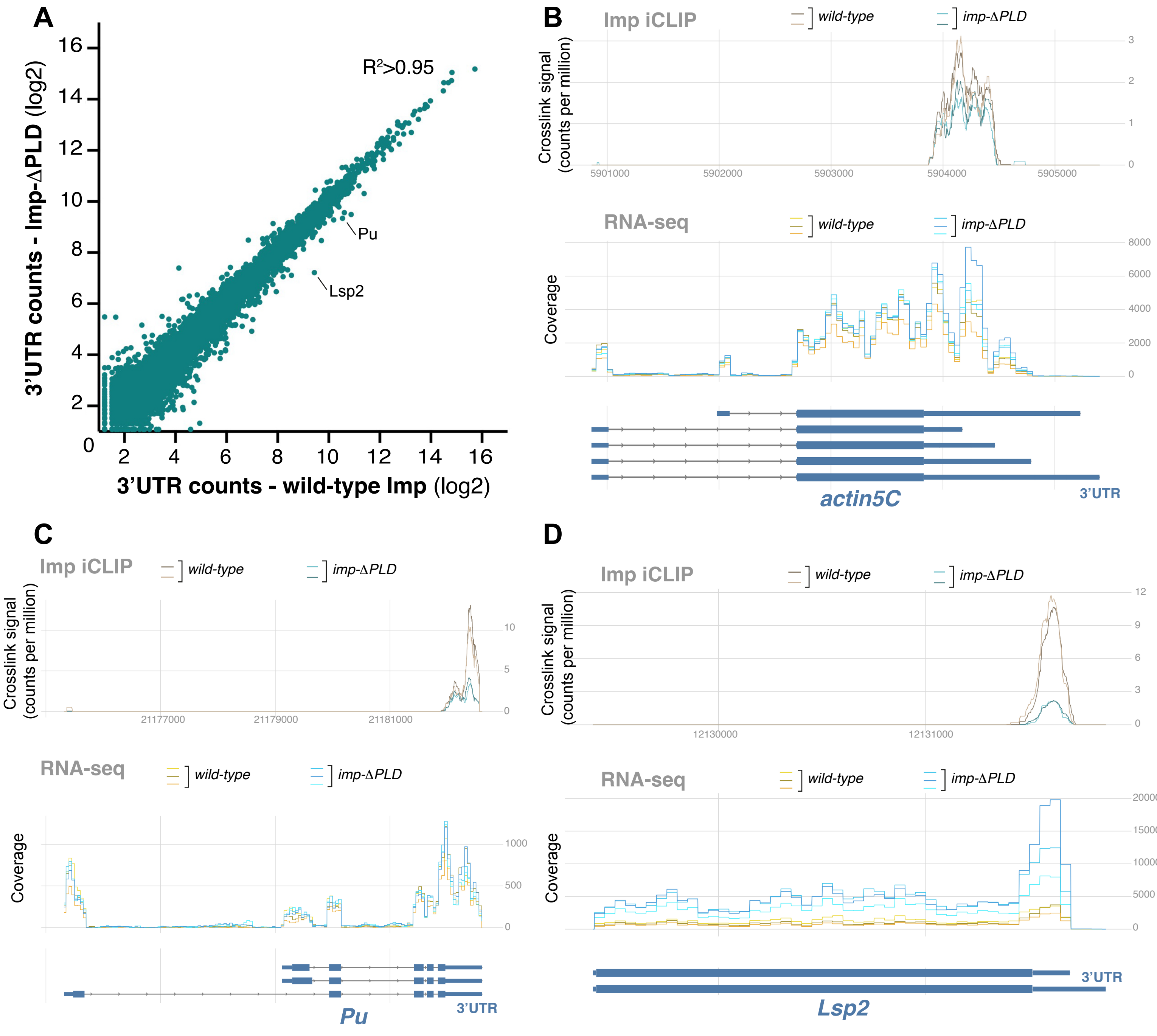
A
R²>0.95
Pu
Lsp2
3'UTR counts - Imp-ΔPLD (log2)
3'UTR counts - wild-type Imp (log2)
B
Imp iCLIP
wild-type
imp-ΔPLD
Crosslink signal (counts per million)
RNA-seq
Coverage
actin5C
3'UTR
C
Imp iCLIP
wild-type
imp-ΔPLD
RNA-seq
Pu
3'UTR
D
Imp iCLIP
wild-type
imp-ΔPLD
RNA-seq
Lsp2
3'UTR

Figure S6

Figure S6. Comparison of Imp wild-type and Imp-ΔPLD iCLiP profiles

(A) Correlation plot showing the normalized counts of 3'UTR-mapped reads obtained for individual RNAs in wild-type (x axis) and Imp-ΔPLD (y axis) iCLIP experiments (same as Figure 6A). The position of the *Pu* and *Lsp2* RNAs are indicated. **(B-D)** Profiles of the Imp wild-type and Imp-ΔPLD iCLIP signals (top, two replicates for each condition) and corresponding input RNA-seq signals (middle, three replicates for each condition) along the *actin5C* (B), *Pu* (C) *and Lsp2* (D) gene regions. Profiles were generated using the *clipplotr* tool [76] and its smoothening function. Intronic and exonic sequences are represented at the bottom by single lines and boxes respectively (large boxes for coding exons and smaller boxes for UTRs).

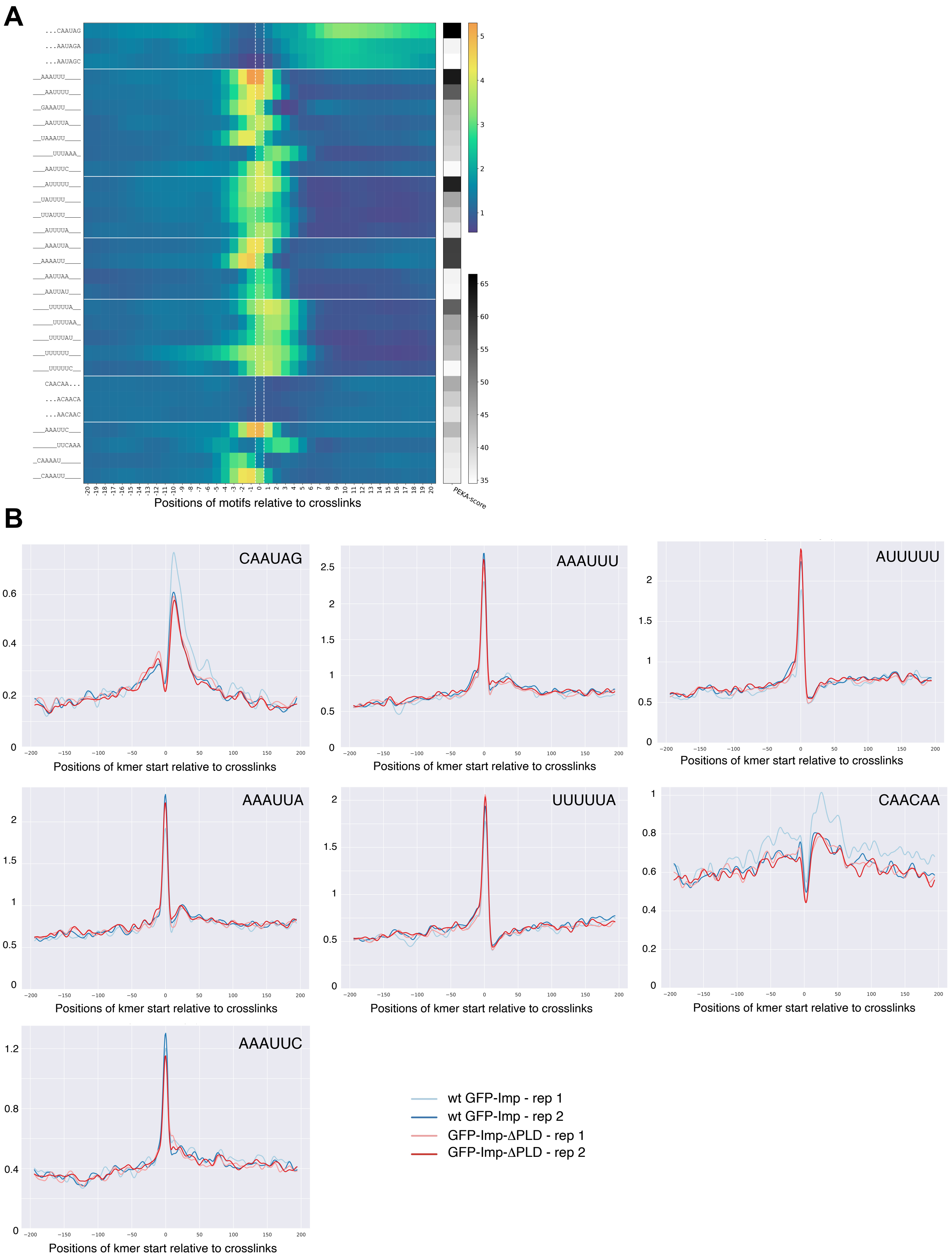
A
Positions of motifs relative to crosslinks
PEKA-score
B
CAAUAG
AAAUUU
AUUUUU
AAAUUA
UUUUUA
CAACAA
AAAUUC
% coverage
Positions of kmer start relative to crosslinks
wt GFP-Imp - rep 1
wt GFP-Imp - rep 2
GFP-Imp-ΔPLD - rep 1
GFP-Imp-ΔPLD - rep 2

Figure S7

Figure S7. Imp preferential motif recognition is not altered in *imp-ΔPLD* mutants.

(A) Heatmap representing the 30 most enriched 6-mers found at wild-type Imp thresholded cross-linked sites in 3'UTRs (as defined by the PEKA algorithm). **(B)** Coverage of 6-mer motifs preferentially recognized by Imp in the two wildtype (wt) and two Imp-ΔPLD replicates (rep 1 and rep 2). Profiles were plotted using a 400 nt-wide window (-200 nt; + 200 nt) around cross-linked sites found in 3'UTRs (aligned at the 0 position). One of the wild-type replicates showed a slightly increased binding to CAA-containing motifs, but this most likely is of technical nature, as this replicate had low cDNA counts and was of thus of lower quality. Thus, no significant changes in the motifs recognized by Imp were observed upon removal of Imp PLD.

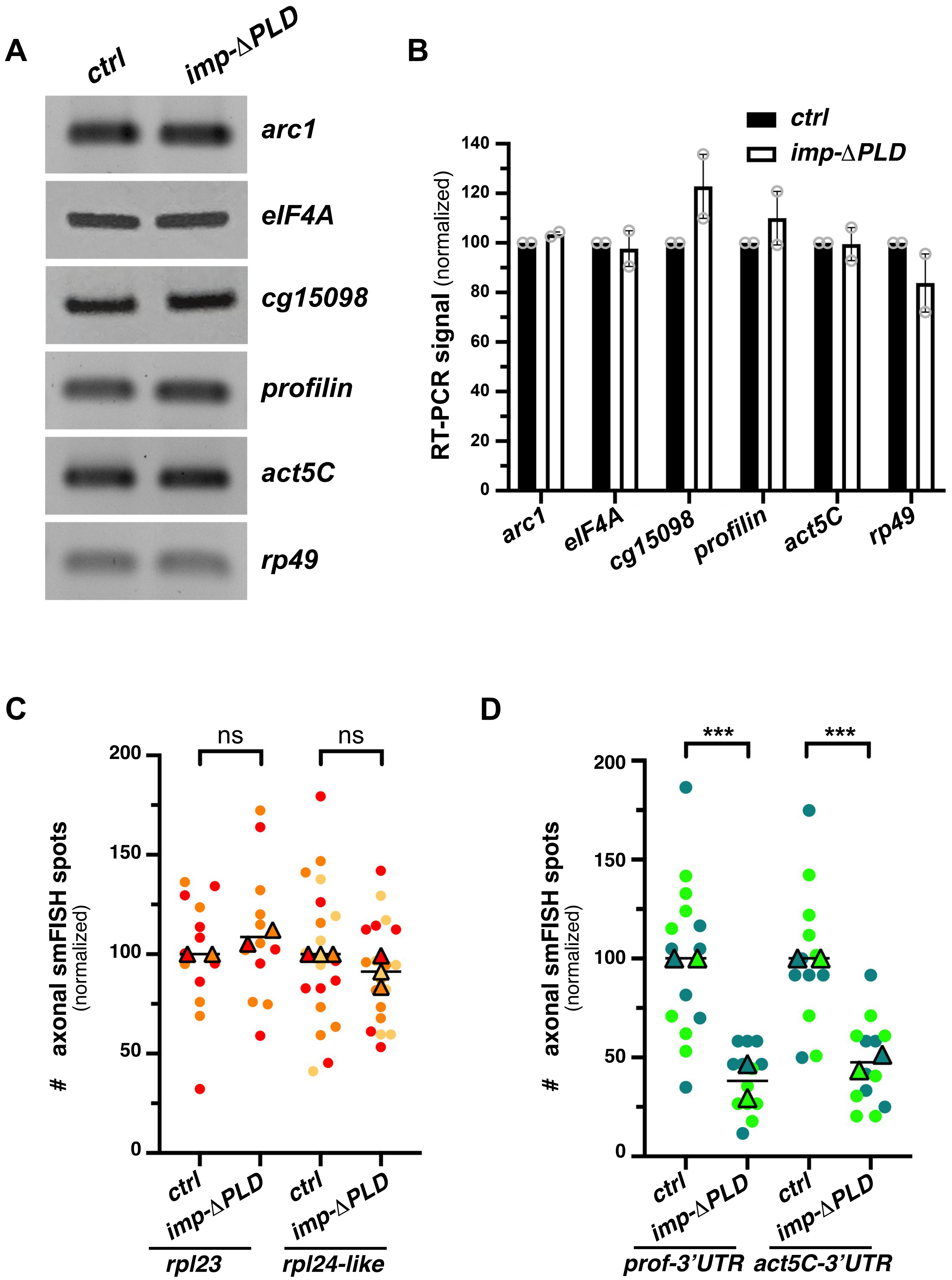
A
ctrl
imp-ΔPLD
arc1
eIF4A
cg15098
profilin
act5C
rp49
B
ctrl
imp-ΔPLD
RT-PCR signal (normalized)
140
120
100
80
60
40
20
0
arc1
eIF4A
cg15098
profilin
act5C
rp49
C
ns
ns
axonal smFISH spots (normalized)
200
150
100
50
0
ctrl
imp-ΔPLD
ctrl
imp-ΔPLD
rpl23
rpl24-like
D
***
***
axonal smFISH spots (normalized)
200
150
100
50
0
ctrl
imp-ΔPLD
ctrl
imp-ΔPLD
prof-3'UTR
act5C-3'UTR

Figure S8

Figure S8. RNA levels and reporter RNA localization in *imp*-Δ*PLD* mutants

(A) Semi-quantitative RT-PCR amplification of *arc1*, *eiF4A*, *cg15098*, *profilin* (*prof*), *actin5C* (*act5C*) and *rp49* from control and *imp-ΔPLD* brain lysates. *rp49* is used as a reference RNA. **(B)** Quantification of the RT-PCR signals. Signals were normalized to the control value for each RNA. Two independent biological replicates were performed. **(C)** Normalized numbers of smFISH spots detected in MB γ axons of wild-type and *imp*-Δ*PLD* mutants. Three replicates were performed and the mean value of each is indicated as a triangle. Data points were color-coded based on the replicate they belong to. At least 12 brains were analyzed per condition. n.s. stands for not significant (Mann-Whitney tests). **(D)** Normalized numbers of *gfp-profilin* 3'UTR (left) and *gfp-actin5C* 3'UTR (right) smFISH spots detected in the axons of control (ctrl) and *imp*-Δ*PLD* MB γ neurons. Two replicates were performed and the mean value of each is indicated as a triangle. Data points were color-coded based on the replicate they belong to. ***, $P<0.001$ (Mann-Whitney tests). Source data are provided as a Source Data file.

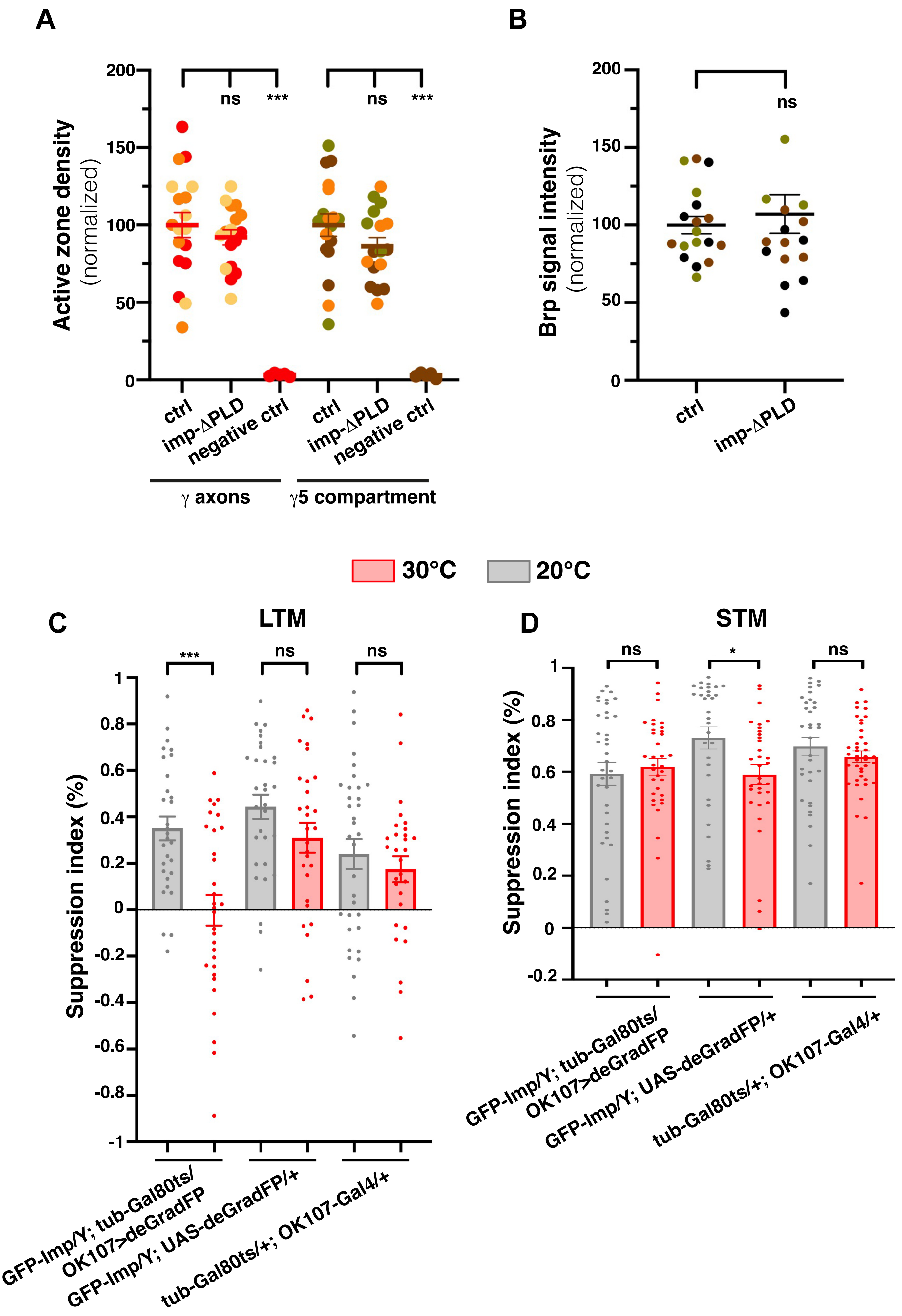
A
B
Active zone density
(normalized)
200
150
100
50
0
ns
***
ctrl
imp-ΔPLD
negative ctrl
γ axons
γ5 compartment
Brp signal intensity
(normalized)
30°C
20°C
C
LTM
D
STM
Suppression index (%)
1
0.8
0.6
0.4
0.2
0
-0.2
-0.4
-0.6
-0.8
-1
*
GFP-Imp/Y; tub-Gal80ts/
OK107>deGradFP
GFP-Imp/Y; UAS-deGradFP/+
tub-Gal80ts/+; OK107-Gal4/+

Figure S9

Figure S9. ***Imp*** **function is required in adult Mushroom Bodies for establishment of LTM.**

(A,B) Normalized numbers (A) and mean intensities (B) of active zones in MB γ axons (left) and MB γ5 compartment in control (ctrl) and *imp-ΔPLD* mutants. Active zones were labeled with anti-Brp antibodies. Negative controls correspond to control brains for which anti-Brp antibodies were omitted. Three replicates were performed and data points color-coded based on the replicate they belong to. At least 16 brains were analyzed per condition except for the negative controls where n=5. n.s. stands for not significant. ***, *P*<0.001 (Kruskall-Wallis test with Dunn's post-tests). **(C,D)** Courtship suppression indices reflecting the Long-Term Memory (LTM, C) and Short-Term Memory (STM, D) performances of mutant flies conditionally expressing the deGradFP construct (GFP-Imp/Y; tub-Gal80ts/+; OK107>deGradFP) and control flies (GFP-Imp/Y; UAS-deGradFP/+ or tub-Gal80ts/+; OK107-Gal4/+). The G080-GFP-Imp protein-trap insertion was used in combination with Gal4-inducible deGradFP nanobodies that target GFP-containing proteins to degradation. Flies were raised at 20°C, a temperature at which the Gal80ts inhibitor is active and thus GFP-Imp degradation blocked. Upon eclosion, half of the flies were shifted to 30°C (red), a temperature at which Gal80ts is inactivated and GFP-Imp protein degraded. OK107-Gal4 was used to drive expression of the deGradFP nanobodies in MB neurons. GFP-Imp/Y; tub-Gal80ts/+; OK107>deGradFP: n= 37 (20°C, STM), 33 (30°C, STM), 30 (20°C, LTM) and 31 (30°C, LTM); GFP-Imp/Y; UAS-deGradFP/+: n= 31 (20°C, STM), 33 (30°C, STM), 31 (20°C, LTM) and 30 (30°C, LTM); tub-Gal80ts/+; OK107-Gal4/+: n= 34 (20°C, STM), 40 (30°C, STM), 34 (20°C, LTM) and 29 (30°C, LTM). **, P*<0.05*; ***, P*<0.001 (unpaired t-tests). n.s. stands for not significant. Source data are provided as a Source Data file.

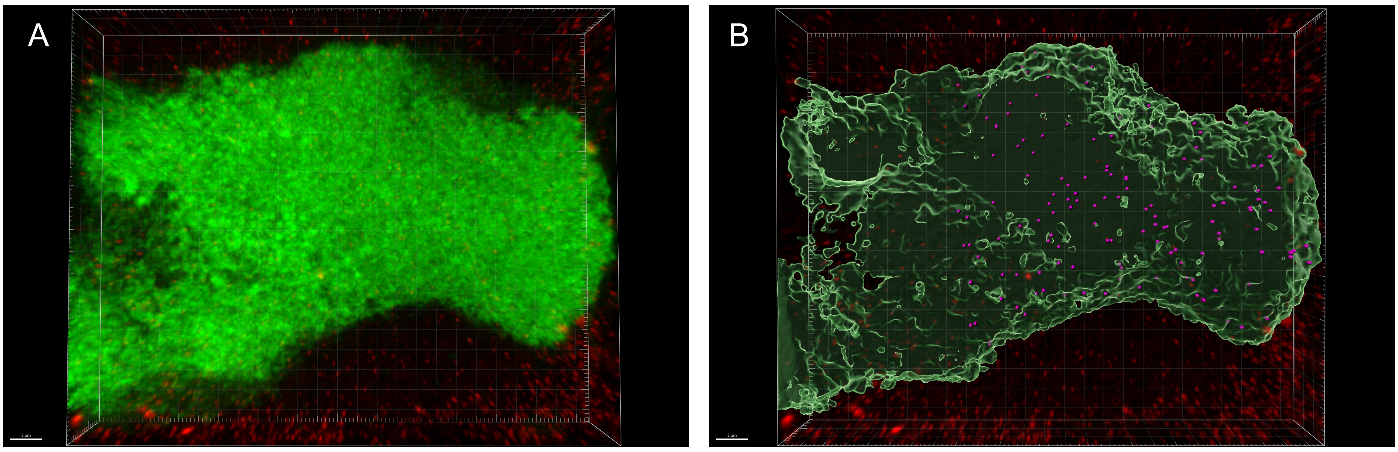

Figure S10

Figure S10. Quantification of smFISH spots.

(A) Maximum Intensity Projection of a confocal volume used for quantification. GFP (green) is expressed under the control of the 201Y-Gal4 driver to label the MB γ lobe. smFISH spots (here obtained using the smiFISH approach) are shown in red. **(B)** Screenshot of smFISH spot detection in Imaris. The surface of MB γ lobe is detected in green, smFISH spots detected within the γ lobe volume are shown in magenta.

a-GFP

GFP-Imp

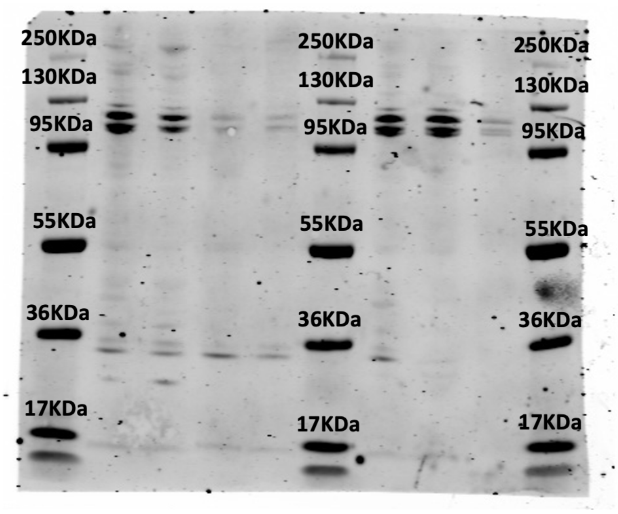

GFP-DImp

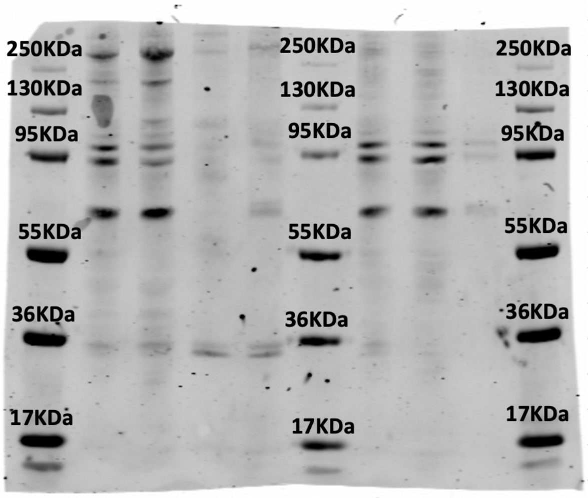

Source data for Figure S3B
