# Supplementary material for: Axonal RNA localization is essential for long-term memory
Source: Nat Commun. 2025 Mar 15;16:2560. doi: 10.1038/s41467-025-57651-7 (PMC11910521; doi:10.1038/s41467-025-57651-7)
Supplement: Supplementary file 2 — Description of Additional Supplementary Files [file 41467_2025_57651_MOESM2_ESM.pdf]

Description of Additional Supplementary Files

File Name: Supplementary Data 1
Description: **Transcripts identified in the synaptosome fraction.**
All transcripts with *Padj*=0.85 (when compared to input samples) are listed in the second sheet. Two-sided Wald tests were used by DESeq2 for hypothesis testing. *Padj* values were calculated by DESeq2 using BenjaminiHochberg false discovery rate (FDR).

File Name: Supplementary Data 2
Description: **GO component categories enriched in the synaptosome fraction.**
Genes included in the different categories are listed in column J. Enrichment was calculated in Gorilla, using standard one-tailed hypergeometric tests and default parameters. FDR was used for multiple comparison.

File Name: Supplementary Data 3
Description: **Table summarizing the behavior of mRNAs mentioned in this study.**

File Name: Supplementary Data 4
Description: **RNAs identified in the Imp RIP-chip experiment.**
RNAs highlighted in grey are represented more than once in the table. Two replicates were considered for the analysis (rep 2 and rep3).

File Name: Supplementary Data 5
Description: **RNAs bound by wild-type Imp in the iCLIP experiment.**
RNAs with at least one significant peak supported by at least 5 cDNAs were considered as Imp-bound. Peak calling and score calculations were performed after pooling replicates 1 and 2. Note that some peaks hit two different genes.

File Name: Supplementary Data 6
Description: **Differential RNA binding in wild-type and Imp-DPLD iCLIP.**
Reads mapping to 3'UTR sequences were used for the DESeq2 analysis. Two-sided Wald tests were used by DESeq2 for hypothesis testing. Padj values were calculated by DESeq2 using BenjaminiHochberg false discovery rate (FDR).

File Name: Supplementary Data 7
Description: **Sequences of the probe sets used for smFISH experiments.**
